# Association of Cerebral Small Vessel Disease Burden and Health-Related Quality of Life after Acute Ischemic Stroke

**DOI:** 10.3389/fnagi.2017.00372

**Published:** 2017-11-13

**Authors:** Yan Liang, Yang-Kun Chen, Min Deng, Vincent C. T. Mok, De-Feng Wang, Gabor S. Ungvari, Chiu-wing W. Chu, Akane Kamiya, Wai-Kwong Tang

**Affiliations:** ^1^Department of Psychiatry, The Chinese University of Hong Kong, Hong Kong, China; ^2^Department of Neurology, Dongguan People’s Hospital, Dongguan, China; ^3^Department of Imaging and Interventional Radiology, The Chinese University of Hong Kong, Hong Kong, China; ^4^Department of Medicine and Therapeutics, The Chinese University of Hong Kong, Hong Kong, China; ^5^Australia/Marian Centre, University of Notre Dame, Perth, WA, Australia; ^6^Department of Rehabilitation, Sagamihara Minami Hospital, Sagamihara, Japan; ^7^Shenzhen Research Institute, The Chinese University of Hong Kong, Shenzhen, China

**Keywords:** cerebral small vessel disease, white matter hyperintensities, lacune, cerebral microbleed, enlarged perivascular space, quality of life, stroke

## Abstract

**Objective**: Cerebral small vessel disease (SVD) is associated with increased mortality, disability and cognitive decline, depression in stroke survivors. This study examined the association between SVD burden, defined by a combination of SVD markers, and health-related quality of life (HRQoL) in acute ischemic stroke.

**Methods**: Patients admitted with acute ischemic stroke of any etiology were prospectively screened between January 2010 to December 2014 and enrolled in the study if they met study entry criteria. HRQoL was evaluated with the 12-item Stroke Specific Quality of Life (SSQoL) at 3 months after the onset of acute ischemic stroke. SVD was ascertained by the presence of any of the SVD markers including lacune, white matter hyperintensities (WMH), cerebral microbleeds (CMB) and enlarged perivascular spaces (EPVS) in the basal ganglia or their combinations on brain magnetic resonance imaging (MRI). The presence of each individual marker scored 1 point and was summed up to generate an ordinal “SVD score” (0–4) capturing total SVD burden. Linear regression was used to determine the associations between SVD burden and HRQoL.

**Results**: Of the743 acute ischemic stroke patients that formed he study sample (mean age: 66.3 ± 10.6 years; 41.7% women), 49.3%, 22.5%, 16.0%, 9.2% and 3.1% had SVD scores of 0, 1, 2, 3 and 4, respectively. After adjusting for demographic, clinical and imaging variables, the SVD score was independently associated with lower overall score of SSQoL (*B* = −1.39, SE = 0.56, *p* = 0.01), and its domains of mobility (*B* = −0.41, SE = 0.10, *p* < 0.001) and vision (*B* = −0.12, SE = 0.06, *p* = 0.03). Acute infract volume (*B* = −1.44, SE = 0.54, *p* = 0.01), functional independence (*B* = 5.69, SE = 0.34, *p* < 0.001) and anxious (*B* = −1.13, SE = 0.23, *p* < 0.001) and depressive symptoms (*B* = −3.41, SE = 0.22, *p* < 0.001) were also the significant predictors of the overall score of SSQoL.

**Conclusion**: The brain’s SVD burden predicts lower HRQoL, predominantly in domains of mobility and vision at 3 months after acute ischemic stroke. The evaluation of SVD burden could facilitate developing individual treatment strategies.

## Introduction

Cerebral small vessel disease (SVD) is a condition of clinical, neuroimaging and neuropathological presentations that causes the damage to the brain’s small perforating vessels (Wardlaw et al., 2013a). The markers of SVD on magnetic resonance imaging (MRI) are white matter hyperintensities (WMH), lacunes, cerebral microbleeds (CMB) and enlarged perivascular spaces (EPVS; Wardlaw et al., 2013a). As these markers are closely associated with increased mortality (Song et al., 2017), poor physical function (Arauz et al., 2003; Charidimou et al., 2016; Sato et al., 2016; Senda et al., 2016; Yang et al., 2016; Helenius et al., 2017), stroke recurrence (Arauz et al., 2003; Lau et al., 2017), cognitive decline (Huijts et al., 2013; Uiterwijk et al., 2016) and depression (Zhang et al., 2016) after stroke, SVD is thought to be a poor prognostic marker of stroke (Kim and Lee, 2015).

Health-related quality of life (HRQoL) is an outcome measure encompassing both physical and mental health. HRQoL is one of the common measure of stroke outcome (Ali et al., 2013). Understanding the factors influencing HRQoL after stroke may help to guide rehabilitation strategies for stroke survivors. Associations between WMH (Tang et al., 2013), CMB (Tang et al., 2011) and lower HRQoL have been reported in stroke survivors, indicating that SVD may have an impact on poststroke HRQoL. The common limitation of all the above studies on SVD is that they have not examined the total SVD burden. A “total SVD score” is constructed by summing up all individual SVD markers into a single measure that is thought to better represent the total brain damage from SVD (Staals et al., 2014). Several recent studies have suggested a role for SVD burden in predicting mortality (Song et al., 2017), unfavorable functional outcome (Yang et al., 2016; Arba et al., 2017), cognitive decline (Huijts et al., 2013; Uiterwijk et al., 2016) and depression (Zhang et al., 2016) after stroke. However, to the best of our knowledge, the association between SVD burden and poststroke HRQoL has never been investigated, which gave the impetus to examine this relationship at 3 months after the index acute ischemic stroke in a large cohort of systematically screened and assessed patients.

## Materials and Methods

### Study Sample

Patients with first-ever or recurrent acute ischemic stroke of any etiology admitted to the Stroke Unit of Prince of Wales Hospital between January 2010 and December 2014 were consecutively screened for the following study entry criteria: having MRI scans on admission, Cantonese speakers and of Chinese ethnicity, having sufficient language, auditory and visual abilities to allow the assessments, having no history of neurological diseases, alcoholism, dementia or severe comorbid medical diseases and no recurrence of the index stroke before the 3-month follow up.

The study protocol was approved by the Joint Chinese University of Hong Kong-New Territories East Cluster Clinical Research Ethic Committee. The consents obtained from all the participants were both informed and written.

### Collection of Demographic and Clinical Data

Within 2 days of admission for the index stroke, a research nurse collected basic demographic data (age, sex and years of education) and medical history including hypertension, diabetes mellitus, hyperlipidemia and previous stroke and assessed the stroke severity using the National Institutes of Health Stroke Scale (NIHSS). Three months after the index stroke, the Chinese version of the Stroke Specific Quality of Life (SSQoL) questionnaire, a valid scale specifically developed to measure poststroke HRQoL (Wang et al., 2003), was administered to all patients. The SSQoL comprises 49 items covering 12 domains, with each item rated on a 5-point Likert scale ranging from “completely true” to “not true at all”. Total and domain scores were generated with a higher score indicating better health condition. The total score ranges from 49 to 245 while the domain scores vary from 3 to 30. The Barthel Index (BI), Lubben Social Network Scale (LSNS), Mini-Mental State Examination (MMSE), Hospital Anxiety Depression Scale-Anxiety Subscale (HADS-AS) and the 15-item Geriatric Depression Scale (GDS) were used to assess functional independence, social support, cognitive function and anxiety and depressive symptoms at the 3-month follow-up, respectively.

### Brain MRI Acquisition

All patients were scanned on a 1.5-T MRI system (Sonata; Siemens Medical) within 1 week of the index admission. The following sequences were performed: diffusion-weighted imaging, gradient echo, T1- and T2-weighted and fluid attenuated inversion recovery. Diffusion-weighted imaging spin-echo echo-planar imaging (long repetition time [TR]/echo delay times [TE]/excitation = 180/122/4; matrix = 128 × 128; field of view (FOV) = 230 mm; slice thickness/gap = 5 mm/1 mm; echo-planar imaging factor = 90; acquisition time = 55 s) with three orthogonally applied gradients was used with b values of 1000 and 500. Axial gradient echo images were acquired as the second sequence, with imaging parameters of TR/TE/excitation = 350/30/2; flip angle of 30°; slice thickness/gap = 5 mm/0.5 mm; FOV = 230 mm; matrix 256 × 256; and acquisition time = 5 min and 4 s. Axial SE T1 (TR/TE/excitation = 425/14/2; FOV = 23 0 mm; slice thickness/gap = 5 mm/0.5 mm; matrix = 256 × 256; and acquisition time = 4 min and 28 s). Turbo spin-echo T2 images were also acquired (TR/TE/excitation = 2500/120/1; turbo factor of 15; FOV = 230 mm; slice thickness/gap = 5 mm/0.5 mm; matrix of 256 × 256; and acquisition time = 1 min and 39 s) and axial FLAIR sequences (TR/TE/inversion-time/excitation = 9000/117/2500/2; turbo factor of 31; FOV = 230 mm; slice thickness/gap = 5 mm/1 mm; matrix = 256 × 256; acquisition time = 3 min and 20 s).

### SVD Total Score

All MRI scans were reviewed by a qualified neurologist (YL) blinded to all other data. The neuroimaging markers of SVD were all defined according to the STRIVE guideline (Wardlaw et al., 2013a). Deep WMH and periventricular WMH were assessed with the Fazekas scale ranging from 0 to 3 (Deep WMH: 0 = no lesions, 1 = punctate foci, 2 = beginning confluency of foci, 3 = large confluent areas; Periventricular WMH: 0 = no lesions, 1 = caps or thin line, 2 = smooth halo, 3 = extension to white matter; Fazekas et al., 1987). Extensive WMH was defined if deep WMH were scored 2 or 3, or periventricular WMH were scored 3. The severity of EPVS in the basal ganglia was rated according to the number of spaces in a slice from one side containing the maximum amount of EPVS: 0 = none; 1 = 1–10; 2 = 11–20; 3 = 21–40; 4 = >40 (Potter et al., 2015). The number of the CMB and lacune were recorded. Furthermore, an ordinal scale validated by Staals et al (Staals et al., 2014) was adopted to calculate the total SVD burden. Briefly, one point was awarded if any lacune or CMB were present, or EPVS in basal ganglia scored 2–4, or extensive WMH (respectively. The four subscores were then summed up to generate a total SVD score that ranged from 0 to 4.

### Other Brain MRI Data

Brain infarcts were detected on T1-weighted images presenting with signals similar to cerebrospinal fluid and diameters exceeding 3 mm. The volumes of infarcts was measured, and the contour of the acute infarcts with restricted water diffusion (identified on diffusion-weighted imaging with b values of 1000) were manually drawn. The total volume was generated by multiplying the total area by the sum of the slice thickness and gap. The locations (cortical, subcortical and infratentorial) of acute infarct and the presence of old infarct were also recorded.

### Statistical Analysis

Data are presented as means ± standard deviations, medians (interquartile ranges or ranges), or proportions as appropriate. Multivariate linear regression analyses were used to investigate the associations between SVD burden and overall SSQoL and its domains adjusting for age, sex, education, history of stroke, hypertension, diabetes mellitus and hyperlipidemia, scores of NIHSS on admission, BI, HADS-AS, GDS, MMSE, acute infarcts’ volume, acute infarcts’ locations (cortical, subcortical and infratentorial), presence of old infarcts with a forward selection method. Due to the skewness of acute infarcts’ volume, they were categorized into four degrees based on the quartile (Degree 1: = 0 mL; Degree 2: 0.01–0.50 mL; Degree 3: 0.51–1.76 mL; Degree 4: >1.76 mL; Lin et al., 2017) and then made them enter all the regression models. The models did not involve any multicollinearity since all predictors showed a variance inflation factor of <10. IBM SPSS Statistics, Version 20.0 software was used for all the above analyses. The level of significance was set at 0.05 (two-tailed).

## Results

### Characteristics of the Study Sample

**Figure 1:**
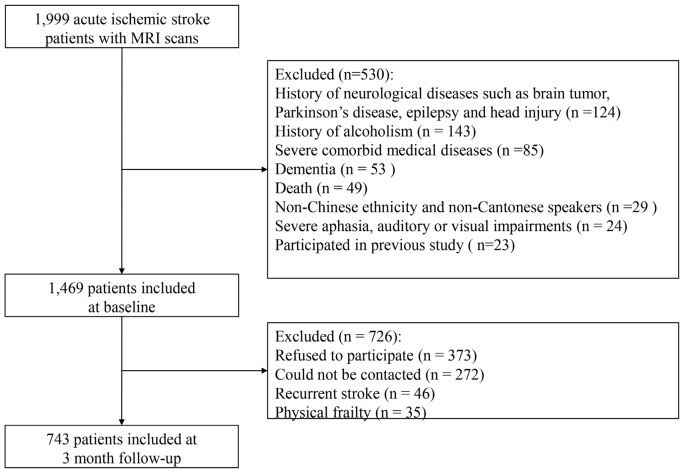
Flow chart of recruitment.

The recruitment flow chart is shown in Figure 1. Only 1999 (35.1%) patients of all admissions with acute ischemic stroke during the study period received MRI scans due to limited access to the MRI machine. Eventually, 743 of these 1999 patients met the inclusion criteria and comprised the final sample. The age did not differ between the included and excluded patients (66.3 ± 10.6 vs. 67.0 ± 13.1; *p* = 0.200), but the excluded patients were more likely to be female (61.3% vs. 38.7%; *p* = 0.043) and had more severe stroke indicated by higher NIHSS scores on the index admission (3 (1–6) vs. 3 (1–5); *p* < 0.001).

The demographic, clinical and MRI characteristics of the whole sample are detailly listed in Table 1. Patients had milder anxious (HAD-AS: 0 (0–3)) and depressive (GDS: 2 (1–5)) symptoms, while relatively good functional independence (BI: 19.1 ± 2.5) and cognitive function (MMSE: 26.9 ± 3.2) at 3 months after stroke. Almost half of the patients did not present with any acute infarct or had a low acute infarct volume (≤0.50 mL). About half of the study sample presented without SVD or with a low SVD burden (SVD score = 0). Of those who had moderate to severe SVD burden (SVD score > 0), 22.5%, 16.0%, 9.2% and 3.1% patients had a SVD score of 1, 2, 3 and 4, respectively (Table 1).

**Table 1:** Demographic, clinical and magnetic resonance imaging (MRI) characteristics of the study sample at the 3 months follow up poststroke.

Characteristics	*N* = 743
Age, years, mean ± SD	66.3 ± 10.6
Women, *n* (%)	310 (41.7)
Education, years, mean ± SD	6.7 ± 4.4
Married, *n* (%)	565 (76.0)
Previous stroke, *n* (%)	75 (10.2)
Hypertension, *n* (%)	486 (66.4)
Diabetes mellitus, *n* (%)	217 (29.6)
Hyperlipidemia, *n* (%)	296 (40.4)
NIHSS score on admission, median (IQR)	3 (1–5)
BI score, mean ± SD	19.1 ± 2.5
LSNS score, mean ± SD	28.5 ± 8.9
HADS-AS score, median (IQR)	0 (0–3)
GDS score, median (IQR)	2 (1–5)
MMSE score, mean ± SD	26.9 ± 3.2
**Acute infarct volume groups**	
Degree 1 (= 0 mL)	249 (33.5)
Degree 2 (0.10–0.50 mL)	122 (16.4)
Degree 3 (0.51–1.76 mL)	185 (24.9)
Degree 4 (>1.76 mL)	187 (25.2)
Cortical acute infarct, *n* (%)	143 (19.2)
Subcortical acute infarct, *n* (%)	284 (38.2)
Infratentorial acute infarct, *n* (%)	118 (15.9)
Presence of old infarct, *n* (%)	153 (20.6)
**Markers of SVD**	
Lacune, *n* (%)	127 (17.1)
Extensive WMH, *n* (%)	276 (37.1)
Moderate to severe EPVS in the basal ganglia, *n* (%)	258 (34.7)
CMB, *n* (%)	91 (12.2)
SVD score 0, *n* (%)	366 (49.3)
SVD score 1, *n* (%)	167 (22.5
SVD score 2, *n* (%)	119 (16.0)
SVD score 3, *n* (%)	68 (9.2)
SVD score 4, *n* (%)	23 (3.1)
Duration from stroke onset to follow-up, days, median (IQR)	104 (94–127)

*BI, Barthel Index; CMB, cerebral microbleeds; EPVS, enlarged perivascular space; HADS-AS, Hospital Anxiety and Depression Scale-Anxiety Subscale; IQR, interquartile range; LSNS, Lubben Social Network Scale; MMSE, Mini-Mental State Examination; NIHSS, National Institutes of Health Stroke Scale; SD, standard deviation; SVD, small vessel diseases; WMH, white matter hyperintensities*.

The SSQoL scores are listed in Table 2. The mean SSQoL total score was 218.8 ± 26.9 with a range of 64–245. Thus, the study sample compromised patients with milder stroke, and relatively favorable clinical outcomes.

**Table 2:** Values of total and dimensional SSQoL scores in the whole study sample (*n* = 743).

	Mean ± SD	Median (Range)
SSQoL total score	218.8 ± 26.9	227 (64–245)
Energy	10.3 ± 4.1	12 (3–15)
Family role	13.1 ± 3.1	15 (3–15)
Language	24.3 ± 1.8	25 (5–25)
Mobility	27.5 ± 4.4	30 (6–30)
Mood	22.7 ± 4.6	25 (5–25)
Personality	12.5 ± 3.5	15 (3–15)
Self-care	24.2 ± 2.6	25 (5–25)
Social role	22.1 ± 5.3	25 (5–25)
Thinking	11.6 ± 3.7	12 (3–15)
Upper extremity function	24.0 ± 2.9	25 (5–25)
Vision	14.3 ± 1.7	15 (3–15)
Work	12.2 ± 3.5	13 (3–15)

*SSQoL, Stroke-Specific Quality of Life*.

### Association between SVD and SSQoL

The associations between SVD burden and overall SSQoL and its domains were estimated with multivariate regression models adjusted for demographic, clinical and imaging characteristics (Table 3 and Supplementary Table S1). The models in which SVD score was significantly associated with SSQoL are displayed in Table 3. The SVD score was a significant predictor of the SSQoL total score (*B* = −1.39, SE = 0.56, *p* = 0.01, Model 1), and domain scores of mobility (*B* = −0.49, SE = 0.10, *p* < 0.001, Model 2) and vision (*B* = −0.12, SE = 0.06, *p* = 0.03, Model 3) after adjusting for confounding factors, with a *R*^2^ of 0.63, 0.53, and 0.10 for Model 1, 2 and 3, respectively (Table 3). SVD burden negatively affected the overall SSQoL, and the domains of mobility and vision. Some of the individual SVD markers, namely extensive WMH (*B* = −3.02, SE = 1.33, *p* = 0.02) and moderate to severe EPVS (*B* = −3.29, SE = 1.37, *p* = 0.02) were showed to be significant predictors of SSQoL, whilst CMB (*p* = 0.05) and lacunes (*p* = 0.77) did not showed significant associations with SSQoL (data not presented).

**Table 3:** Multivariate linear models on the association between SVD burden and the SSQoL.

	Model 1: SSQoL Total^a^			Model 2: SSQoL Mobility^a^			Model 3: SSQoL Vison^a^		
	*B*	SE	*p*	*B*	SE	*p*	*B*	SE	*p*
Functional independence (BI)	5.69	0.34	<0.001	1.36	0.06	<0.001	0.14	0.03	<0.001
Anxious symptoms (HADS-AS)	−1.13	0.23	<0.001	−	−	−	−0.05	0.02	0.02
Depressive symptoms (GDS)	−3.41	0.22	<0.001	−0.13	0.04	<0.001	−0.07	0.02	<0.001
Acute infract volume	−1.44	0.54	0.01	−	−	−	−	−	−
Infratentorial acute infarct	−	−	−	−0.67	0.32	0.04			
SVD burden (SVD score)	−1.39	0.56	0.01	−0.49	0.10	<0.001	−0.12	0.06	0.03
*R*^2^	0.63			0.53			0.10		

*BI, Barthel Index; HADS-AS, Hospital Anxiety and Depression Scale-Anxiety Subscale; MMSE, Mini-Mental State Examination; NIHSS, National Institute of Health Stroke Scale; SE, standard error; SSQoL, Stroke-Specific Quality of Life; SVD, small vessel diseases. ^a^Model 1, 2 and 3 were multivariate linear regressions models adjusted for age, sex, education, history of stroke, hypertension, diabetes mellitus and hyperlipidemia, NIHSS on admission, BI, HADS-AS, GDS, MMSE, acute infarct volume, acute infarct locations (cortical, subcortical and infratentorial), presence of old infarct and SVD score with forward selection method (only the significant predictors were listed here)*.

In conclusion, only WMH and EPVS showed significant association with HRQoL when the markers were considered separately; however, SVD burden was a significant predictor of HRQoL when the four individual markers were combined into a “SVD score”.

### Association between Other Characteristics and SSQoL

The associations between each domain of SSQoL and all the potential predictors are demonstrated in Supplementary Table S1. Functional independence (BI), depressive (GDS) and anxiety symptoms (HADS-AS) were the three most consistent predictors of both overall SSQoL and its domains (Table 1; Supplementary Table S1). Acute infarcts’ volume was significantly associated with reduced overall SSQoL (*B* = −1.39, SE = 0.56, *p* = 0.01; Model 1, Table 1), and the domains of family-role (*B* = −0.20, SE = 0.09, *p* = 0.03; Model 2, Supplementary Table S1) and social-role (*B* = −0.36, SE = 0.15, *p* = 0.02; Model 8, Supplementary Table S1). Cortical acute infarcts were associated with mood (*B* = −0.64, SE = 0.31, *p* = 0.04; Model 5, Supplementary Table S1), whereas infratentorial acute infarcts correlated with overall SSQoL (*B* = −1.39, SE = 0.56, *p* = 0.01; Model 1, Table 3), the energy (*B* = −1.00, SE = 0.40, *p* = 0.01; Model 1, Supplementary Table S1), mobility (*B* = −0.67, SE = 0.32, *p* = 0.04; Model 4, Supplementary Table S1) and thinking domain scores (*B* = −0.24, SE = 0.11, *p* = 0.03; Model 9, Supplementary Table S1). Other significant predictors of domain specific SSQoL were age, female sex and hypertension (Supplementary Table S1). In short, the overall or domain scores of SSQoL were affected by a wide range of demographic, clinical and MRI factors.

## Discussion

The main finding of this study is that SVD burden capturing the total brain damage resulting from SVD was independently and negatively associated with HRQoL, particularly in the mobility and vison domains. To the best of our knowledge, this was the first study that investigated the association between SVD burden and stroke survivors’ HRQoL.

The main findings of this study—SVD burden and the individual SVD markers of WMH and EPVS are predictors of HRQoL—are in line with earlier results that lobar CMB (Tang et al., 2011) and WMH (Tang et al., 2013) impact on stroke survivors’ physical health and overall HRQoL. Further, pre-stroke WMH burden (Senda et al., 2016; Helenius et al., 2017), presence of multiple lacune (Arauz et al., 2003), CMB (Charidimou et al., 2016), or moderate-to-severe EPVS in the basal ganglia (Yang et al., 2016) were all found to be risk factors for functional disability after stroke. A combination of SVD markers better predicted poststroke physical outcome than one marker (Yang et al., 2016; Arba et al., 2017). Arba et al. (2017) reported that a SVD score, a combination of WMH, lacunes and brain atrophy based on computerized tomography scans, was associated with disability at 90 days after stroke in patients receiving intravenous thrombolysis. However, none of the above studies combined all 4 SVD markers based on MRI to evaluate the total brain damage caused by SVD. More recently, similar to our rating method on SVD burden, a prospective study of lacunar stroke demonstrated the role of SVD burden in depression at 3 months after stroke (Zhang et al., 2016). Another two large-scale studies of patients with acute ischemic stroke found that SVD burden could be an imaging marker predicting mortality after stroke (Song et al., 2017) and stroke recurrence (Lau et al., 2017). This study extended the above findings by considering all the markers of SVD and the domain-specific HRQoL.

Several lines of evidence could explain the negative effect of SVD burden on overall HRQoL and mobility. Pre-existing SVD has been suggested to be a marker of brain frailty to ischemia, as SVD markers indicates accumulating pathologic injuries in the brain’s small perforating vessels (Wardlaw et al., 2013b). At the immediate onset of acute ischemia, a more extensive WMH increases the infarcts’ volume by aggravating the systemic inflammation or hypercoagulability triggered by ischemia (Helenius et al., 2017). A high CMB burden increases the risk of developing symptomatic hemorrhages after revascularization (Tsivgoulis et al., 2016). Moreover, a greater SVD burden might slow down the brain’s recovery from stroke. The brain’s ability to recover is related to the integrity of neural networks. In the context of extensive SVD, the structural network in the white matter is disrupted thus impairing the brain’s plasticity and compensatory mechanisms (Kim et al., 2015; Helenius et al., 2017). From a clinical point of view, stroke patients with a higher SVD burden are therefore more predisposed to functional disabilities and are less likely to recover from stroke.

Visual functions have been rarely investigated in stroke. An association between SVD burden and the visual functioning was found in this study. Consistent with this finding, visual impairments commonly occur in cerebral autosomal dominant arteriopathy presenting with subcortical infarcts and leukoencephalopathy (CADASIL), an inherited adult-onset disease of cerebral SVD. Retinal microvascular abnormalities are also commonly reported in patients with sporadic cerebral SVD (Kwa et al., 2002). In a cross-sectional study of patients with type 2 diabetes, a close association between diabetic retinopathy and WMH or lacunes was observed (Sanahuja et al., 2016). The above findings support the hypothesis that retinal microvascular diseases and cerebral SVD are two different manifestations originated from one common pathologic entity sharing similar risk factors (Kwa et al., 2002). These findings could explain why cerebral SVD burden negatively affected domains of vision of SSQoL in the current study.

About half of this study sample had low SVD burden, similar to the figure of 45%–50% reported in two cohort studies on all subtypes of acute ischemic stroke (Lau et al., 2017; Song et al., 2017). However, in the lacunar stroke population, the SVD burden was higher with just 30% of subjects with a SVD score of 0 (Huijts et al., 2013). SVD burden is related to a slightly higher risk for recurrent stroke in stroke accounted to SVD compared to other etiologies (Lau et al., 2017). It is, therefore, possible that the impact of SVD burden on poststroke HRQoL may vary across the different subtypes of acute ischemic stroke. However, the current study lacked such information on the etiologic subtypes of ischemeic stroke and therefore was unable to delineate the contribution of SVD burden in HRQoL by stroke subtype.

In the present study, functional independence, anxiety and depressive symptoms were found to be predictors of HRQoL, a finding that has been consistently reported (Raju et al., 2010; Sprigg et al., 2013). Of note, the size and locations of acute infarct was significantly associated with one or more domains of HRQoL in this study. Consistently, the acute infarcts’ volume was recently found to contribute modestly to the prediction of domains of applied cognition–general concerns of HRQoL, however, this study did not explore the locations of acute infarct (Lin et al., 2017). Our study showed that the infratentorial acute infarct could negatively affect several domains of SSQoL, implicating a necessity of incorporation of the locations of infarcts or other brain lesions when investigating their impacts on HRQoL after stroke.

Several strengths and weaknesses of the present study should be addressed. The major strength is the large, carefully selected, homogenous sample. Additionally, this study utilized comprehensive information about SVD by combining all 4 neuroimaging markers into a single “SVD score”. A wide range of confounding factors were considered including demographic, clinical and the characteristics of infarcts. However, the first major limitation is that the sample comprised patients mainly with mild and moderate stroke, among whom a large proportion had a SVD score of 0. This might limit the generalizability of the results to more severely affected patients. Second, due to insufficient clinical information, the diagnosis of stroke subtypes was not considered in the analysis. Lastly, the study evaluated patients’ HRQoL after the relatively short post-stroke interval of 3 months, thereby failing to elucidate the long-term impact of SVD on the HRQoL.

In conclusion, this study found that SVD burden, a combination of four SVD markers, predicted lower HRQoL, particularly the dimensions of mobility and vision examined at 3 months after acute ischemic stroke. The findings suggest that screening baseline SVD burden might help to identify stroke patients with poor poststroke HRQoL, particularly in the physical and visual functioning domains, who would benefit from more focused treatment and rehabilitation strategies. Further studies are warranted to validate these findings and investigate the long-term impact of SVD burden on poststroke HRQoL.

## Author Contributions

W-KT and YL made substantial contributions to the conception and design of the work. YL, Y-KC, MD, VCTM, D-FW and CWC made substantial contributions to the acquisition of data for the work. YL and Y-KC made substantial contributions to the analysis of data. VCTM, D-FW, CWC, GSU, AK and W-KT contributed to interpretations of data for the work. YL drafted the article, while all remaining authors critically revised the draft for intellectual content. All authors gave their approval of the version to be published, and agreed to be accountable for all aspects of the work in ensuring that questions related to the accuracy or integrity of any part of the work were appropriately investigated and resolved.

## Conflict of Interest Statement

The authors declare that the research was conducted in the absence of any commercial or financial relationships that could be construed as a potential conflict of interest.

## Abbreviations

BI: Barthel Index
CMB: cerebral microbleeds
EPVS: enlarged perivascular spaces
FOV: field of view
GDS: Geriatric Depression Scale
HADS-AS: Hospital Anxiety Depression Scale-Anxiety Subscale
HRQoL: health-related quality of life
IQR: interquartile ranges
LSNS: Lubben Social Network Scale
MMSE: Mini-Mental State Examination
NIHSS: National Institutes of Health Stroke Scale
SD: standard deviations
SSQoL: Stroke Specific Quality of Life
SVD: small vessel disease
TE: echo delay times
TR: repetition time
WMH: white matter hyperintensities.

## References

[B1] Ali M. Fulton R. Quinn T. Brady M. VISTA Collaboration. (2013). How well do standard stroke outcome measures reflect quality of life? A retrospective analysis of clinical trial data. Stroke 44, 3161–3165. 10.1161/STROKEAHA.113.001126 24052510

[B2] Arauz A. Murillo L. Cantu C. Barinagarrementeria F. Higuera J. (2003). Prospective study of single and multiple lacunar infarcts using magnetic resonance imaging: risk factors, recurrence, and outcome in 175 consecutive cases. Stroke 34, 2453–2458. 10.1161/01.str.0000090351.41662.91 14500936

[B3] Arba F. Inzitari D. Ali M. Warach S. J. Luby M. Lees K. R. (2017). Small vessel disease and clinical outcomes after IV rt-PA treatment. Acta Neurol. Scand. 136, 72–77. 10.1111/ane.12745 28233290

[B4] Charidimou A. Shoamanesh A. International META-MICROBLEEDS Initiative. (2016). Clinical relevance of microbleeds in acute stroke thrombolysis: comprehensive meta-analysis. Neurology 87, 1534–1541. 10.1212/wnl.0000000000003207 27629086

[B5] Fazekas F. Chawluk J. B. Alavi A. Hurtig H. I. Zimmerman R. A. (1987). MR signal abnormalities at 1.5 T in Alzheimer’s dementia and normal aging. Am. J. Roentgenol. 149, 351–356. 10.2214/ajr.149.2.351 3496763

[B6] Helenius J. Mayasi Y. Henninger N. (2017). White matter hyperintensity lesion burden is associated with the infarct volume and 90-day outcome in small subcortical infarcts. Acta Neurol. Scand. 135, 585–592. 10.1111/ane.12670 27573379 PMC5332524

[B7] Huijts M. Duits A. van Oostenbrugge R. J. Kroon A. A. de Leeuw P. W. Staals J. (2013). Accumulation of MRI markers of cerebral small vessel disease is associated with decreased cognitive function. A study in first-ever lacunar stroke and hypertensive patients. Front. Aging Neurosci. 5:72. 10.3389/fnagi.2013.00072 24223555 PMC3818574

[B9] Kim H. J. Im K. Kwon H. Lee J. M. Kim C. Kim Y. J.. (2015). Clinical effect of white matter network disruption related to amyloid and small vessel disease. Neurology 85, 63–70. 10.1212/wnl.0000000000001705 26062629 PMC4501945

[B8] Kim B. J. Lee S. H. (2015). Prognostic impact of cerebral small vessel disease on stroke outcome. J. Stroke 17, 101–110. 10.5853/jos.2015.17.2.101 26060797 PMC4460329

[B10] Kwa V. I. van der Sande J. J. Stam J. Tijmes N. Vrooland J. L. Amsterdam Vascular Medicine Group. (2002). Retinal arterial changes correlate with cerebral small-vessel disease. Neurology 59, 1536–1540. 10.1212/01.wnl.0000033093.16450.5c 12451193

[B11] Lau K. K. Li L. Schulz U. Simoni M. Chan K. H. Ho S. L.. (2017). Total small vessel disease score and risk of recurrent stroke: validation in 2 large cohorts. Neurology 88, 2260–2267. 10.1212/wnl.0000000000004042 28515266 PMC5567324

[B12] Lin C. Lee J. Chatterjee N. Corado C. Carroll T. Naidech A.. (2017). Predicting domain-specific health-related quality of life using acute infarct volume. Stroke 48, 1925–1931. 10.1161/strokeaha.117.017094 28536175 PMC5505231

[B13] Potter G. M. Chappell F. M. Morris Z. Wardlaw J. M. (2015). Cerebral perivascular spaces visible on magnetic resonance imaging: development of a qualitative rating scale and its observer reliability. Cerebrovasc. Dis. 39, 224–231. 10.1159/000375153 25823458 PMC4386144

[B14] Raju R. S. Sarma P. S. Pandian J. D. (2010). Psychosocial problems, quality of life, and functional independence among Indian stroke survivors. Stroke 41, 2932–2937. 10.1161/strokeaha.110.596817 20966411

[B15] Sanahuja J. Alonso N. Diez J. Ortega E. Rubinat E. Traveset A.. (2016). Increased burden of cerebral small vessel disease in patients with type 2 diabetes and retinopathy. Diabetes Care 39, 1614–1620. 10.2337/dc15-2671 27281772

[B16] Sato S. Delcourt C. Heeley E. Arima H. Zhang S. Al-Shahi Salman R.. (2016). Significance of cerebral small-vessel disease in acute intracerebral hemorrhage. Stroke 47, 701–707. 10.1161/strokeaha.115.012147 26846860

[B17] Senda J. Ito K. Kotake T. Kanamori M. Kishimoto H. Kadono I.. (2016). Association of leukoaraiosis with convalescent rehabilitation outcome in patients with ischemic stroke. Stroke 47, 160–166. 10.1161/strokeaha.116.012885 26658442

[B18] Song T. J. Kim J. Song D. Yoo J. Lee H. S. Kim Y. J.. (2017). Total cerebral small-vessel disease score is associated with mortality during follow-up after acute ischemic stroke. J. Clin. Neurol. 13, 187–195. 10.3988/jcn.2017.13.2.187 28406586 PMC5392462

[B19] Sprigg N. Selby J. Fox L. Berge E. Whynes D. Bath P. M. (2013). Very low quality of life after acute stroke: data from the efficacy of nitric oxide in stroke trial. Stroke 44, 3458–3462. 10.1161/strokeaha.113.002201 24149005

[B20] Staals J. Makin S. D. Doubal F. N. Dennis M. S. Wardlaw J. M. (2014). Stroke subtype, vascular risk factors, and total MRI brain small-vessel disease burden. Neurology 83, 1228–1234. 10.1212/wnl.0000000000000837 25165388 PMC4180484

[B21] Tang W. K. Chen Y. K. Lu J. Ahuja A. T. Chu W. C. Mok V. C.. (2011). Cerebral microbleeds and quality of life in acute ischemic stroke. Neurol. Sci. 32, 449–454. 10.1007/s10072-011-0571-y 21479609

[B22] Tang W. K. Liang H. J. Chen Y. K. Ahuja A. T. Chu W. C. Mok V. C.. (2013). White matter hyperintensities and quality of life in acute lacunar stroke. Neurol. Sci. 34, 1347–1353. 10.1007/s10072-012-1267-7 23247600

[B23] Tsivgoulis G. Zand R. Katsanos A. H. Turc G. Nolte C. H. Jung S.. (2016). Risk of symptomatic intracerebral hemorrhage after intravenous thrombolysis in patients with acute ischemic stroke and high cerebral microbleed burden: a meta-analysis. JAMA Neurol. 73, 675–683. 10.1001/jamaneurol.2016.0292 27088650

[B24] Uiterwijk R. van Oostenbrugge R. J. Huijts M. De Leeuw P. W. Kroon A. A. Staals J. (2016). Total cerebral small vessel disease MRI score is associated with cognitive decline in executive function in patients with hypertension. Front. Aging Neurosci. 8:301. 10.3389/fnagi.2016.00301 28018214 PMC5149514

[B29] Wang Y. Ma J. Li J. (2003). The study on reliability, validity and responsiveness of the Chinese version of stroke-specific quality of life. Chin. J. Geriatr. Heart. Brain. Vessel. Dis. 5, 391–394.

[B26] Wardlaw J. M. Smith E. E. Biessels G. J. Cordonnier C. Fazekas F. Frayne R.. (2013a). Neuroimaging standards for research into small vessel disease and its contribution to ageing and neurodegeneration. Lancet Neurol. 12, 822–838. 10.1016/S1474-4422(13)70124-8 23867200 PMC3714437

[B25] Wardlaw J. M. Smith C. Dichgans M. (2013b). Mechanisms of sporadic cerebral small vessel disease: insights from neuroimaging. Lancet Neurol. 12, 483–497. 10.1016/s1474-4422(13)70060-7 23602162 PMC3836247

[B27] Yang H. Shen R. Jin Z. Li J. Wu Y. Xu Y.. (2016). Dilated virchow-robin spaces in first-ever lacunar stroke patients: topography and clinical correlations. J. Stroke Cerebrovasc. Dis. 25, 306–311. 10.1016/j.jstrokecerebrovasdis.2015.09.034 26521169

[B28] Zhang X. Tang Y. Xie Y. Ding C. Xiao J. Jiang X.. (2016). Total magnetic resonance imaging burden of cerebral small-vessel disease is associated with post-stroke depression in patients with acute lacunar stroke. Eur. J. Neurol. 24, 374–380. 10.1111/ene.13213 27933697

