# Supplementary material for: Association of Cerebral Small Vessel Disease Burden and Health-Related Quality of Life after Acute Ischemic Stroke
Source: Front Aging Neurosci. 2017 Nov 13;9:372. doi: 10.3389/fnagi.2017.00372 (PMC5693845; doi:10.3389/fnagi.2017.00372)
Supplement: Supplementary file 1 [file Table_1.docx]

Table e-1. The associations between demographic, clinical and imaging characteristics and domains of SSQoL using multivariate linear regressions

|  | **Model 1: Energy** | | | **Model 2: Family role** | | | **Model 3: language** | | | **Model 4: Mobility** | | | **Model 5: Mood** | | | **Model 6: Personality** | | |
| --- | --- | --- | --- | --- | --- | --- | --- | --- | --- | --- | --- | --- | --- | --- | --- | --- | --- | --- |
| **Predictors** | **B** | **SE** | **p** | **B** | **SE** | **p** | **B** | **SE** | **p** | **B** | **SE** | **p** | **B** | **SE** | **p** | **B** | **SE** | **p** |
| Age |  |  |  |  |  |  | 0.02 | 0.01 | 0.001 |  |  |  |  |  |  |  |  |  |
| Female sex | -0.69 | 0.30 | 0.02 |  |  |  |  |  |  |  |  |  | 0.61 | 0.26 | 0.02 | 0.64 | 0.27 | 0.02 |
| Education, (years) |  |  |  |  |  |  |  |  |  |  |  |  |  |  |  |  |  |  |
| Previous stroke |  |  |  |  |  |  |  |  |  |  |  |  |  |  |  |  |  |  |
| Hypertension |  |  |  |  |  |  |  |  |  |  |  |  |  |  |  |  |  |  |
| Diabetes mellitus |  |  |  |  |  |  |  |  |  |  |  |  |  |  |  |  |  |  |
| Hyperlipidemia |  |  |  |  |  |  |  |  |  |  |  |  |  |  |  |  |  |  |
| NIHSS |  |  |  |  |  |  |  |  |  |  |  |  |  |  |  |  |  |  |
| BI | 0.18 | 0.08 | 0.02 | 0.22 | 0.06 | <0.001 | 0.17 | 0.03 | <0.001 | 1.38 | 0.06 | <0.001 | 0.32 | 0.07 | <0.001 |  |  |  |
| HADS-AS | -0.24 | 0.05 | <0.001 |  |  |  |  |  |  |  |  |  | -0.24 | 0.05 | <0.001 | -0.31 | 0.05 | <0.001 |
| GDS | -0.42 | 0.05 | <0.001 | -0.37 | 0.03 | <0.001 | -0.04 | 0.02 | 0.05 | -0.13 | 0.04 | <0.001 | -0.76 | 0.04 | <0.001 | -0.24 | 0.04 | <0.001 |
| MMSE |  |  |  |  |  |  | 0.05 | 0.02 | 0.02 |  |  |  |  |  |  |  |  |  |
| Old infarcts |  |  |  |  |  |  |  |  |  |  |  |  |  |  |  |  |  |  |
| Acute infarcts’ volume |  |  |  | -0.20 | 0.09 | 0.03 |  |  |  |  |  |  |  |  |  |  |  |  |
| Cortical acute infarcts |  |  |  |  |  |  |  |  |  |  |  |  | -0.64 | 0.31 | 0.04 |  |  |  |
| Subcortical acute infarcts |  |  |  |  |  |  |  |  |  |  |  |  |  |  |  |  |  |  |
| Infratentorial acute infarcts | -1.00 | 0.40 | 0.01 |  |  |  |  |  |  | -0.67 | 0.32 | 0.04 |  |  |  |  |  |  |
| **SVD score** |  |  |  |  |  |  |  |  |  | **-0.49** | **0.10** | **<0.001** |  |  |  |  |  |  |
| **R^2^** | 0.27 |  |  | 0.24 |  |  | 0.09 |  |  | 0.53 |  |  | 0.52 |  |  | 0.18 |  |  |

|  | **Model 7: Self-care** | | | **Model 8: Social role** | | | **Model 9: Thinking** | | | **Model 10: upper**  **extremity function** | | | **Model 11: vision** | | | **Model 12: Work** | | |
| --- | --- | --- | --- | --- | --- | --- | --- | --- | --- | --- | --- | --- | --- | --- | --- | --- | --- | --- |
| **Predictors** | **B** | **SE** | **p** | **B** | **SE** | **p** | **B** | **SE** | **p** | **B** | **SE** | **p** | **B** | **SE** | **p** | **B** | **SE** | **p** |
| Age |  |  |  |  |  |  | 0.02 | 0.01 | <0.001 |  |  |  |  |  |  |  |  |  |
| Female sex |  |  |  |  |  |  |  |  |  |  |  |  | 0.61 | 0.26 | 0.02 | 0.64 | 0.27 | 0.02 |
| Education, per year |  |  |  |  |  |  |  |  |  |  |  |  |  |  |  |  |  |  |
| Previous stroke |  |  |  |  |  |  |  |  |  |  |  |  |  |  |  |  |  |  |
| Hypertension |  |  |  |  |  |  | -0.58 | 0.28 | 0.04 |  |  |  |  |  |  |  |  |  |
| Diabetes mellitus |  |  |  |  |  |  |  |  |  |  |  |  |  |  |  |  |  |  |
| Hyperlipidemia |  |  |  |  |  |  |  |  |  |  |  |  |  |  |  |  |  |  |
| NIHSS |  |  |  |  |  |  |  |  |  |  |  |  |  |  |  |  |  |  |
| BI | 0.90 | 0.03 | <0.001 | 0.63 | 0.10 | <0.001 | 0.16 | 0.03 | <0.001 | 1.07 | 0.04 | <0.001 | 0.14 | 0.03 | <0.001 |  |  |  |
| HADS-AS |  |  |  |  |  |  | -0.18 | 0.05 | <0.001 |  |  |  | -0.05 | 0.02 | 0.02 | -0.31 | 0.05 | <0.001 |
| GDS | -0.06 | 0.02 | 0.001 | -0.57 | 0.05 | <0.001 | -0.39 | 0.04 | <0.001 | -0.08 | 0.02 | <0.001 | -0.07 | 0.02 | 0.001 | -0.24 | 0.04 | <0.001 |
| MMSE |  |  |  |  |  |  | 0.12 | 0.04 | 0.003 |  |  |  |  |  |  |  |  |  |
| Old infarcts |  |  |  |  |  |  |  |  |  |  |  |  |  |  |  |  |  |  |
| Acute infarcts’ volume |  |  |  | -0.36 | 0.15 | 0.02 |  |  |  |  |  |  |  |  |  |  |  |  |
| Cortical acute infarcts |  |  |  |  |  |  |  |  |  |  |  |  |  |  |  |  |  |  |
| Subcortical acute infarcts |  |  |  |  |  |  |  |  |  |  |  |  |  |  |  |  |  |  |
| Infratentorial acute infarcts |  |  |  |  |  |  | -0.24 | 0.11 | 0.03 |  |  |  |  |  |  |  |  |  |
| **SVD score** |  |  |  |  |  |  |  |  |  |  |  |  | **-0.12** | **0.06** | **0.03** |  |  |  |
| R^2^ | 0.58 |  |  | 0.28 |  |  | 0.23 |  |  | 0.59 |  |  | 0.10 |  |  | 0.18 |  |  |

BI=Barthel Index; GDS= Geriatric Depression Scale; HADS-AS=Hospital Anxiety and Depression Scale-Anxiety Subscale; MMSE=Mini-Mental State Examination; NIHSS=National Institutes of Health Stroke Scale; SE=standard error; SSQoL= Stroke-Specific Quality of Life.
